# ‘I wasn't made to feel like a nut case after all’: A qualitative story completion study exploring healthcare recipient and carer perceptions of good professional caregiving relationships

**DOI:** 10.1111/hex.13871

**Published:** 2023-10-19

**Authors:** Rebecca Feo, Jessica A. Young, Kristi Urry, Michael Lawless, Sarah C. Hunter, Alison Kitson, Tiffany Conroy

**Affiliations:** ^1^ College of Nursing and Health Sciences Flinders University Adelaide South Australia Australia; ^2^ Caring Futures Institute Flinders University Adelaide South Australia Australia; ^3^ School of Psychology University of Adelaide Adelaide South Australia Australia

**Keywords:** carer, conceptualisations, healthcare recipient, patient–provider relationships, perceptions, professional caregiving relationships, qualitative story completion

## Abstract

**Background:**

Professional caregiving relationships are central to quality healthcare but are not always developed to a consistently high standard in clinical practice. Existing literature on what constitutes high‐quality relationships and how they should be developed is plagued by dyadic conceptualisations; discipline, context and condition‐specific research; and the absence of healthcare recipient and informal carer voices. This study aimed to address these issues by exploring how healthcare recipients and carers conceptualise good professional caregiving relationships regardless of discipline, care setting and clinical condition.

**Design:**

A qualitative story completion approach was used. Participants completed a story in response to a hypothetical stem that described a healthcare recipient (and, in some instances, carer) developing a good relationship with a new healthcare provider. Stories were analysed using reflexive thematic analysis.

**Participants:**

Participants were 35 healthcare recipients and 37 carers (*n* = 72 total).

**Results:**

Participants' stories were shaped by an overarching discourse that seeking help from new providers can elicit a range of unwanted emotions for both recipients and carers (e.g., anxiety, fear, dread). These unwanted emotions were experienced in relation to recipients' presenting health problems as well as their anticipated interactions with providers. Specifically, recipient and carer characters were fearful that providers would dismiss their concerns and judge them for deciding to seek help. Good relationships were seen to develop when healthcare providers worked to relieve or minimise these unwanted emotions, ensuring healthcare recipients and carers felt comfortable and at ease with the provider and the encounter. Participants positioned healthcare providers as primarily responsible for relieving recipients' and carers' unwanted emotions, which was achieved via four approaches: (1) easing into the encounter, (2) demonstrating interest in and understanding of recipients' presenting problems, (3) validating recipients' presenting problems and (4) enabling and respecting recipient choice. Participants' stories also routinely oriented to temporality, positioning relationships within recipients' and carers' wider care networks and biographical and temporal contexts.

**Conclusion:**

The findings expand our understanding of professional caregiving relationships beyond dyadic, static conceptualisations. Specifically, the findings suggest that high‐quality relationships might be achieved via a set of core healthcare provider behaviours that can be employed across disciplinary, context and condition‐specific boundaries. In turn, this provides a basis to support interprofessional education and multidisciplinary healthcare delivery, enabling different healthcare disciplines, specialties, and teams to work from the same understanding of what is required to develop high‐quality relationships.

**Patient or Public Contribution:**

The findings are based on stories from 72 healthcare recipient and carer participants, providing rich insight into their conceptualisations of high‐quality professional caregiving relationships.

## 1 INTRODUCTION

Caregiving relationships within professional healthcare settings (often termed patient–provider relationships) are typically defined as therapeutic relationships that enable planning, delivery and evaluation of care to meet healthcare recipients' health and wellbeing needs.^1^ A growing body of work demonstrates the importance of these relationships for healthcare quality and safety as well as healthcare experiences and outcomes.^2^, ^3^, ^4^ For healthcare recipients, high‐quality professional caregiving relationships help them to feel safe, confident in, and satisfied with their care and can facilitate effective behaviour change, symptom resolution and pain reduction, as well as enhanced functional health, quality of life and emotional well‐being.^3^, ^4^, ^5^, ^6^, ^7^ In turn, these positive outcomes can reduce unnecessary healthcare utilisation and costs.^4^ For healthcare providers, developing professional and therapeutic relationships with the people they care for has been shown to positively impact work satisfaction and morale,^4^, ^8^ which has implications for workforce retention and turnover.^9^ In turn, several discipline‐specific standards and codes of conduct emphasise that developing relationships with healthcare recipients is a core tenet of meeting people's unique health and care needs.^10^, ^11^, ^12^ The importance of high‐quality relationships is also recognised in broader healthcare theories, models and frameworks, including person‐centred care,^13^ the Caring Life Course Theory,^14^ relationship‐centred care^15^ and fundamental care.^16^

Despite the recognised importance of professional caregiving relationships at theoretical and policy levels, and the growing evidence to demonstrate their impact, the recognised importance of these relationships is not matched by the rigour with which they are conceptualised, nor is it readily translated into everyday practice, including in clinical practice guidelines.^1^, ^17^, ^18^ This is partly because theories and policy discourse offer little guidance on how relationships are best developed and maintained in practice.^19^ Additionally, the evidence base for methods and interventions to facilitate and sustain high‐quality professional caregiving relationships is lacking.^18^, ^20^, ^21^ It is perhaps unsurprising then that these relationships are often poorly developed and experienced by healthcare providers, healthcare recipients and informal carers (e.g., family).^22^, ^23^, ^24^, ^25^ Poor‐quality professional caregiving relationships are key contributors to an acknowledged ‘crisis of care’ globally^26^ and commensurate public and academic concerns about healthcare providers' capacity to deliver quality care^27^ and health systems' commodification of care in the quest for efficient, cost‐effective service delivery.^28^, ^29^

While the reasons for poor‐quality professional caregiving relationships are complex, we explicate two interrelated issues pertinent to our study and which are relevant across disciplines, care settings and clinical conditions. The first issue concerns how professional caregiving relationships are researched. Studies often explore relationships within specific disciplines, care settings, patient groups or clinical conditions only. While there is some utility in this (e.g., in building discipline‐specific knowledge), it runs the risk of perpetuating siloed approaches to care delivery. As a result, conceptualisations of professional caregiving relationships are often highly idiosyncratic to different disciplines, care settings and conditions. This then has implications for interprofessional education and multidisciplinary care because healthcare providers from different disciplines and specialities might not be working from the same understanding of what is required to achieve high‐quality caregiving relationships. Furthermore, many studies have privileged theoretical, researcher and, to a lesser extent, provider perspectives (for similar arguments, see^1^, ^7^, ^18^, ^30^, ^31^, ^32^). Yet, evidence indicates that recipients' and carers' understandings of relationships can provide additional, valuable information and are often more predictive of clinical outcomes.^30^, ^31^

The second issue concerns whether and how the role of informal carers is conceptualised within professional caregiving relationships. Despite increased recognition that these relationships occur within complex care networks involving multiple stakeholders,^14^, ^33^, ^34^ conceptual models and measurement and intervention approaches often view relationships as dyadic (e.g., provider–recipient or provider–carer only) (for similar arguments, see^18^, ^21^, ^32^, ^35^, ^36^, ^37^). The relationships occurring between healthcare providers, recipients and carers are studied in some contexts (e.g., dementia care^38^) but not others,^35^ and there is little consistency in whether and how carers are and should be engaged as partners in care delivery.^21^ Taken together, these issues demonstrate a need to move beyond ‘methodological individualism,^39^ ^,p.2449^ within the study of professional caregiving relationships and focus instead on understanding: (1) the perspectives of all involved in these relationships, particularly recipients and carers; (2) the nature of relationships beyond the dyadic; and (3) relationships beyond discipline‐, context‐, and condition‐specific boundaries. This study, therefore, explores how healthcare recipients and informal carers conceptualise professional caregiving relationships, regardless of healthcare discipline, care setting or clinical condition, focusing specifically on what makes good (i.e., high‐quality) relationships.

## 2 METHODS

### 2.1 Design and methodological framework

This study involved a qualitative approach, informed by critical realism and using story completion as the data collection method. Within story completion, participants are presented with a hypothetical scenario about fictional characters, or a ‘story stem’, and asked to write a story response.^40^, ^41^ Participants' stories provide insight into the sociocultural discourses, assumptions and meaning‐making practices that shape how they understand certain phenomena^42^ and how they behave in the real‐world.^43^ Story completion was chosen for this study because it enables researchers to explore participants' perceptions without the requirement to have had a specific experience.^40^, ^41^, ^42^ Given the variable, sometimes poor, quality of professional caregiving relationships, story completion enabled us to explore possibilities for what makes a good relationship, even if those possibilities have not been experienced by participants.^44^ Furthermore, because story stems are centred on hypothetical characters, story completion permits access to a range of meanings, not just socially desirable ones,^42^ thus reducing participants' attempts to align with researchers' motivations and shape their responses accordingly.^41^ Story completion therefore allowed us to examine a broader range of social and cultural understandings in relation to the topic at hand.^44^

Critical realism was chosen as the methodological framework to guide the study. Critical realism frequently underpins qualitative research,^45^ including story completion studies.^43^, ^46^ Critical realism posits that there is a real social world that can be objectively observed (a realist ontology) but that this observation and the knowledge it generates is inherently shaped by social, historical and cultural discourses (an interpretivist epistemology).^45^, ^47^, ^48^ This framework aligns with the present study in three ways. First, the realist lens enables us to take a pragmatic approach, where the goal is to generate knowledge about real, observable phenomena to inform practice relating to healthcare provision and health behaviours. Second, an interpretivist lens aligns with story completion aim to understand the shared social meanings that shape people's perceptions of real phenomena.^41^ Finally, an interpretivist epistemology acknowledges that researchers have an active role in interpreting research data and shaping research findings, thus aligning with our method for data analysis—reflexive thematic analysis.

### 2.2 Participants

Participants were eligible to take part if they lived in Australia, were aged 18+ years, were fluent in English (reading and writing), and were:

1. Healthcare recipients (i.e., adults who used healthcare services but were not healthcare providers or informal carers); or
2. Informal (i.e., unpaid) carers who were not professional healthcare providers.

Participants were recruited via social media (e.g., Twitter, University alumni webpages), and newsletters and emails from relevant Australian healthcare organisations. Recruitment material included a participant information sheet and a link to the online story completion task. Participants received a $20AUD gift voucher for participation.

### 2.3 Data collection

Data were collected between September 2021 and January 2022. Participants were presented with a story stem depicting a healthcare provider and recipient meeting for the first time and developing a good relationship. Participants were asked to write a story about how this relationship developed. Follow‐up prompts asked participants to elaborate on their stories (e.g., how the characters felt, the behaviours they employed). Participants were randomly assigned one of two stems: a dyadic healthcare encounter, involving a healthcare provider and recipient only; or a triadic encounter, involving a provider, recipient and informal carer (Table 1). The aim of including two stems was to determine if participants' conceptualisations of professional caregiving relationships differed when carers were involved in those relationships. Participants chose their own care setting, healthcare provider discipline, and reason for the encounter (see Urry et al.^44^ for full participant instructions). Participants were also asked demographic information (e.g., gender, age, frequency of contact with the healthcare system).

**Table 1:** Story stems.

Dyadic stem	Today, Lee meets a new healthcare provider (Alex). Lee has never received care from Alex before today. A good relationship develops between Lee and Alex, and the encounter goes well.
Triadic stem	Lee and Robin are both adults. They know each other well, and Robin is also Lee's carer. Today they meet Alex, who is a new healthcare provider for Lee. Lee has never received care from Alex before today. A good relationship develops between Lee, Robin, and Alex, and the encounter goes well.

### 2.4 Data analysis

Participant demographic and story information (i.e., word count) were analysed using descriptive statistics. Story stem responses and responses to follow‐up prompts were analysed using reflexive thematic analysis, which aims to generate patterns of shared meaning through researchers' interpretive processes.^45^ We adopted a six‐stage approach.^49^ The first stage was *familiarisation*. The first, second, and third authors read the data set independently, noting initial ideas, including similarities and differences across participant groups and story stems, and met to discuss their initial impressions. Following this discussion, the second author inductively *generated codes*. Then, to *generate candidate themes and subthemes*, the first and second authors categorised the codes independently and met to discuss categorisations before meeting with the wider team. This stage involved multiple data sessions with all authors and a mapping exercise to understand relationships between codes. The research aim guided our choices around what would generate meaningful patterns, helping to shape the analytic focus. In the fourth and fifth steps—*developing and reviewing candidate themes* and *refining, defining and naming themes*—the first and second authors developed definitions and scope for each theme and subtheme. The final step was the *production of this report*, enabling further refinement of themes and subthemes.

### 2.5 Ethical considerations

The study was approved by the Flinders University Human Research Ethics Committee (ID: 4610). Participation was voluntary. Participants were informed they could withdraw at any time by closing the web page, however, any data provided would be used. Participants completed an electronic consent form on the first page of the online story completion task before participating.

### 2.6 Rigour

Within critical realism and reflexive thematic analysis, rigour comes from researcher reflexivity.^50^, ^51^ We attended to reflexivity in several ways. First, we piloted the story stems and instructions with university colleagues in research and teaching roles to ensure understandability and to identify whether we were biasing participants to respond in particular ways. Following this process, some demographic questions were removed as their relevance to the study aim was not evident. We also piloted the study materials with participants by initially advertising the study through a single recruitment avenue and checking the data from the first five participants. No changes were required to the study materials following this process and the five stories were included in the data set. Second, notes and discussions from familiarisation were considered at all analytic stages to ensure the data guided our analysis. Third, we undertook analysis independently and collaboratively, allowing us to generate a range of ideas and to challenge each other on those ideas and how they related to the research aim.^52^

## 3 FINDINGS

Seventy‐two individuals participated: 35 healthcare recipients (*n* = 21 who completed the dyadic story stem; *n* = 14 the triadic stem) and 37 carers (*n* = 17 dyadic; *n* = 20 triadic). Participants ages ranged from 19 to 79 years (median = 41.5). Most participants identified as women (*n* = 50), lived in urban areas (*n* = 63), and did not have a chronic illness (*n* = 44) or disability (*n* = 54). In the previous 12 months, all but two participants (recipients and carers) had visited a healthcare provider for themselves at least once, with most (*n* = 23) visiting a professional 7–12 times. Participants typically visited general practitioners (*n* = 69), followed by dentists (*n* = 46), medical specialists (*n* = 37), physiotherapists (*n* = 30), optometrists (*n* = 26), psychologists (*n* = 23) and radiographers (*n* = 22). Carer participants cared for 1–4 people, most commonly their parents (*n* = 18), children (*n* = 17), spouse/partner (*n* = 8), friend (*n* = 3) and/or other family member (*n* = 3). In the previous 12 months, most carers (*n* = 20) had visited a healthcare provider 12+ times as part of their carer role.

The median response length (story stem and follow‐up prompts) was 234 words (range: 47–1811). Participants' stories featured different healthcare providers (e.g., general practitioners, personal care workers, allied health professionals), care settings (e.g., general practice clinic, healthcare recipient's home) and reasons‐for‐visit (e.g., physical injury, mental health concerns, personal care support). Most stories (79%) described only the initial meeting of the healthcare provider, recipient and carer (if present), while the rest (21%) took place over multiple encounters. All stories, but one, involved face‐to‐face healthcare encounters and most (93%) were written in the third person.

Our analysis demonstrates that participants' stories were shaped by an overarching discourse that seeking help from, and developing relationships with, new healthcare providers can elicit a range of unwanted emotions for both healthcare recipients and carers (e.g., anxiety, distress, fear, worry, dread). These emotions were experienced in relation to the presenting health problem and/or the anticipated interaction with the provider. Good relationships were seen to develop when healthcare provider characters actively worked to relieve or minimise these unwanted emotions, ensuring healthcare recipients and carers felt comfortable and at ease with the provider and the encounter more generally. This notion of developing high‐quality relationships by relieving unwanted emotions was evident in healthcare recipient and carer stories and across both story stems. Hence, the data from both participant groups and both stems have been considered together.

We explicate two main themes: (1) Meeting new healthcare providers can elicit a range of unwanted emotions for healthcare recipients and carers, and (2) developing good relationships is achieved by relieving or minimising healthcare recipients' and (by proxy) carers' initial, unwanted emotions (Table 2). Illustrative excerpts are presented, labelled by participant group (healthcare recipient or carer), story stem (dyad or triad), and participant number. In extracts, ‘Lee’ is the healthcare recipient character (hereafter ‘recipient’), ‘Robin’ the informal carer character (hereafter ‘carer’), and ‘Alex’ the healthcare provider character (hereafter ‘provider’). We have removed spelling mistakes for readability.

**Table 2:** Description of themes and subthemes.

**Theme 1: Meeting new healthcare providers can elicit a range of unwanted emotions for recipients and carers**.	
*Description: Recipients and carers typically experienced unwanted or undesired emotions at the beginning of a healthcare encounter, which stemmed from a range of sources*.	
Recipients unwanted emotions stemmed from their concerns about (1) their presenting health problem: current poor health; potential assessment or treatment procedures; and (2) interactions with the provider: unfamiliarity of a new provider; anticipated provider invalidation; past negative help‐seeking experiences.	
Carers unwanted emotions stemmed from their concerns about (1) their anticipated role in encounter: describing the presenting problem; and (2) interactions between the provider and recipient: recipient difficulty in adapting to a new provider; providers' (possible) insufficient experience, skills or knowledge.	
**Theme 2: Developing good relationships is achieved by relieving or minimising care recipients' and (by proxy) carers' initial, unwanted emotions**.	
*Description: Recipients' and carers' initial, unwanted emotions were relieved or minimised by four main provider approaches (each involving multiple possible behaviours). These approaches enabled recipients and carers to feel comfortable and at ease with the provider and the encounter more generally, which was constitutive of good relationships*.	
**Subtheme**	**Provider behaviours**
Easing into the encounter	Provided relevant professional information about qualifications and experience.
	Began with ‘small talk’ or general conversation.
	Asked questions to get to know recipients.
Demonstrating interest in and understanding of recipients' presenting health problems	Listened attentively—were quiet when needed and demonstrated attention and interest nonverbally (e.g., through eye contact, nodding).
	Allowed recipients time to explain their concerns without rushing to other aspects of the encounter (e.g., diagnosis).
	Asked relevant follow‐up questions relating to recipients' presenting problems.
Validating recipients' presenting health problems	Showed empathy and understanding for recipients' situations and current concerns.
	Reassured recipients that seeking professional help for their presenting problem is valid.
Enabling and respecting recipient choice	Asked about recipient preferences regarding assessment and treatment and carer presence within the encounter.
	Provided explanations and answered recipient and carer questions using simple, easy‐to‐understand language.
	Provided options rather than directives.
	Respected recipient choices (including refusal of treatment).
	Did not force recipients to make decisions immediately.

### 3.1 Theme 1: Meeting new healthcare providers can elicit a range of unwanted emotions for recipients and carers

Participants often described recipient and carer characters as arriving at the healthcare encounter experiencing anxiety, nerves, discomfort, dread, fear, worry, concern, apprehension, scepticism, hesitance, trepidation and/or wariness. Participants positioned these emotions as unwanted or undesired by the recipient and carer characters and as requiring relief or minimisation. Participants constructed the causes of these emotions as different in nature for recipients and carers.

For recipient characters, their initial emotions arose from two sources. The first related to their presenting problems. Recipients were described as having anxiety or nerves about their current poor health, which prompted them to seek help, and/or about potential undesired assessment or treatment outcomes arising from help‐seeking (e.g., adverse reaction to vaccination, involuntary hospital admission for mental health reasons):

He [Lee] further explained that he was worried that he would have a severe adverse reaction to the Covid vaccine—not necessarily anaphylaxis, but a triggering of his autoimmune disease. (Carer Dyad 03).

The second source of recipients' initial emotions related to their anticipated interactions with providers. Recipients were described as nervous or uncomfortable about having to explain their presenting problem and disclose potentially sensitive information to someone unfamiliar: *What I had to tell him was pretty personal, so my fear was that he wanted to know more about it* (Carer Dyad 10). Additionally, recipients were depicted as anticipating provider invalidation or judgement, often expecting providers to dismiss their concerns and deem their presenting problems as unworthy of professional help:

They're [Lee is] a little nervous about it [seeing a provider] as they just don't feel like there's any major issue and so think the physio[therapist] will probably feel the same. (Healthcare Recipient Dyad 01)

Some participants linked recipients' fear or nerves about the interactional aspects of the encounter, especially fear of invalidation, to previous experiences of dismissive, untrustworthy or judgemental providers. Recipient characters were therefore trepidatious about new providers and sceptical that these relationships would be better than previous ones:

Lee was nervous at first. He'd had previous encounters with medical professionals who were dismissive or who had made up their minds before even entering the room what the outcome would be. (Carer Dyad 03)

Lee was nervous about meeting yet another new psychiatrist, how she will be the same as all others, saying one thing but not to be trusted because she [Lee] had been let down too many times before. (Carer Triad 13)

Recipients' fears of invalidation or judgement and previous negative experiences with providers also posed barriers to timely help‐seeking, with recipients only seeking professional help after experiencing their problems for some time:

Lee would like some help with prolonged mental health issues that have been untreated due to Lee not thinking it was worth a GP's [General Practitioner's] time. (Healthcare Recipient Dyad 08)

Like recipients, carers' initial, unwanted emotions stemmed from two sources. First, carers were worried about their role within the encounter and what might be required of them. Like recipients, carers were wary about the unfamiliarity of a new provider and potential requirements to describe the presenting problem:

Robin and Lee went in together, bracing for the formalities of any first appointment. Trying to summarise a life of complex care and obscure maladies, even with someone who had received a well‐written referral letter, could be draining. (Healthcare Recipient Triad 14)

Second, carers were concerned about the recipient and how the encounter might unfold for them. Carers were worried the recipient might feel uncomfortable receiving care from someone new or take extended time to warm to the provider and adapt to a new relationship: *Robin was sitting on the edge of her seat not knowing how Lee would handle someone new coming into the home routine* (Carer Triad 03). Additionally, carers were concerned that the provider might have insufficient experience, skills, or knowledge to provide appropriate care for the recipient:

Robin was very wary about meeting the new carer and was very keen to check out his qualifications and references to make sure that Lee was well cared for. (Healthcare Recipient Triad 07)

In summary, this theme demonstrates participants' orientation to help‐seeking from, and forming relationships with, new providers as often eliciting a range of unwanted emotions for recipients and carers. For recipient characters, these emotions arose not only from their poor health but from the requirement to describe their problem to someone unfamiliar and from anticipating providers might dismiss their concerns and invalidate their reasons for help‐seeking. While carer characters were similarly concerned about explaining the presenting problem to someone new, they were also concerned for the recipient, including how they might respond to a new provider. As Theme 2 demonstrates, participants oriented to relief from these emotions for both recipients and carers as central to developing, and as evidence of, good professional caregiving relationships.

### 3.2 Theme 2: Developing good relationships is achieved by relieving or minimising recipients' and (by proxy) carers' initial, unwanted emotions

At some point in participants' stories, recipient and carer characters were described as experiencing a relief from or minimisation in their initial, unwanted emotions, enabling them to feel comfortable, relaxed and at ease with the provider and the encounter more generally. This relief, and the subsequent feelings of ease and comfort, were seen as central to and constitutive of the development of good relationships. Providers were constructed as primarily responsible for facilitating relief, which was typically achieved via four approaches: (1) easing into the encounter; (2) demonstrating interest in and understanding of recipients' presenting health problems; (3) validating recipients' presenting health problems; and (4) enabling and respecting recipient choice. Each approach involved multiple possible behaviours, as depicted in Table 2 and the subthemes below.

Provider characters primarily directed these behaviours to recipients, however these behaviours relieved unwanted emotions for both recipients and carers. For recipients, then, relief was achieved directly through their interactions with the provider. By contrast, for carers, relief and therefore relationship development, was achieved indirectly by observing interactions between the provider and recipient and feeling relieved that the recipient was comfortable with the provider. As such, carer characters often had minimal direct involvement in the encounter:

Robin felt slightly guarded in the beginning as he was unfamiliar with Alex. He left the encounter feeling relieved that Lee had warmed to Alex quickly and would be comfortable receiving care from him. (Healthcare Recipient Triad 10)

Robin felt like he/she needed to sit in the background, not say too much until Lee's approval of Alex and then only share information when appropriate. (Healthcare Recipient Dyad 11)

The four subthemes below depict the main approaches provider characters adopted to relieve the unwanted emotions outlined in Theme 1, thus ensuring recipients and, by proxy, carers were comfortable and at ease with the provider and the encounter. The subthemes and illustrative quotes focus primarily on recipient rather than carer characters, reflecting the indirect way in which carers' unwanted emotions were relieved or minimised within participants' stories.

#### 3.2.1 Easing into the encounter

Provider characters routinely started encounters by initiating conversation not explicitly about recipients' presenting problems. This involved the provider (1) informing recipients and carers about their qualifications and experience and how the interaction would unfold (i.e., what to expect), (2) making ‘small talk’ *(Healthcare Recipient Dyad 01, Healthcare Recipient Triad 09)* with recipients and carers and (3) asking the recipient questions to get to know them. Beginning encounters in this way helped recipients to avoid feeling rushed and provided a buffer for the recipient between meeting a new provider and divulging personal, potentially sensitive information. This approach helped relieve initial discomfort around the requirement to disclose personal information to someone unfamiliar. In turn, recipients felt able to disclose relevant information during the encounter and willing to return to the provider for help with the same or a different problem:

He [the provider] called me by my name and explained to me that he was new to the Medical Centre and briefly what his brief working history had been. He asked me if I had any questions about what he had said. I'm glad he didn't just rush through and ask me why I was there because I was feeling a bit anxious about making the appointment in the first place. (Carer Dyad 10)

Lee feels Alex takes the time to get to know them as a person, not just as a patient. Lee therefore feels comfortable going back to see Alex about the presenting issue and to also consult Alex about other health and wellbeing issues. (Carer Dyad 06)

#### 3.2.2 Demonstrating interest in and understanding of recipients' presenting health problems

Participants routinely depicted provider characters as adopting behaviours to demonstrate interest in and understanding of recipients' presenting problems. Specific behaviours included listening attentively, such as by ‘being quiet when [they] needed to be’ (Healthcare Recipient Triad 15), and showing attention and interest nonverbally, such as ‘with smiles, eye contact, nodding’ (Healthcare Recipient Triad 14). Providers also allowed recipients adequate time to explain concerns and asked relevant follow‐up questions to confirm their understanding. Conversations typically occurred between providers and recipients, with carers becoming involved only when providers required additional information or when recipients had communication difficulties. Providers' interest in and understanding of recipients' presenting problems enabled recipients to overcome discomfort about disclosing personal information to someone unfamiliar:

[Lee felt] overwhelmed and anxious to start off with but because of Alex's demeanour and interest Lee felt more at ease and was then able to open up a bit about what she was seeking and why. (Carer Dyad 10)

#### 3.2.3 Validating recipients' presenting health problems

Participants' stories routinely oriented to the importance of providers validating recipients' concerns about their presenting problems, thus relieving recipients' fear that these problems were not worthy of professional help. Providers validated recipients' presenting problems in two ways. First, providers showed empathy and respect for recipients' experiences. Participants typically framed this empathy and respect as the absence of specific behaviours (e.g., not being judgemental, condescending, dismissive, patronising or critical of recipients' concerns), further demonstrating characters' initial fears that providers would demonstrate these behaviours within the encounter:

It was such a relief to actually ask for this help as I had been agonising about it for weeks. It wasn't so bad and I wasn't made to feel like a nut case after all. (Carer Dyad 10)

When Lee sat down anxious, Alex was able to calm Lee down just by being professional. Not judging Lee's answers or questions, not rushing, asking how they were and genuinely caring. It was for these reasons Lee would be back to see Alex. (Healthcare Recipient Dyad 17)

Second, providers explicitly reassured recipients that seeking professional help for their presenting problem was reasonable and even necessary:

Alex attentively listens and validates Lee's concerns regarding their mental health. Lee thanks Alex for listening and Alex reassures Lee that coming to the GP for mental health concerns is extremely valid. (Healthcare Recipient Dyad 08)

Many participants positioned provider validation as enabling recipients to feel ‘heard’, ensuring they were comfortable to return to the provider if needed:

Lee felt very validated all throughout the appointment. They felt heard and it helped them to feel like that they'd done the right thing by coming in and seeking professional help and to feel positive about feeling better soon. It's also made Lee feel very comfortable about the prospect of ever needing to book an appointment for any issue in the future. (Healthcare Recipient Dyad 01)

#### 3.2.4 Enabling and respecting recipient choice

Participants' stories depicted providers as enabling and respecting recipient choice primarily in relation to assessment and treatment but also carer presence within the encounter. Providers enabled choice by: (1) using easy‐to‐understand language to provide explanations and respond to questions, ensuring recipients had sufficient understanding of assessment and treatment options (e.g., risks and benefits); 2) providing ‘gentle suggestions’ (Carer Triad 05) rather than directives; (3) soliciting recipients' opinion and preferences (e.g., ‘Alex asks Lee what he thought could be done to improve the situation’, Carer Triad 05); (4) respecting recipient choices (such as to refuse treatment); and (5) not forcing recipients to make decisions immediately. Enabling and respecting recipient choice helped alleviate recipient fears of certain assessment or treatment procedures, allowing them to be more informed about these procedures and to have greater control over whether and when they took place:

She [Alex] offers that if Lee is uncomfortable with having an internal examination performed on them today, they are welcome to make another appointment, or this can be skipped. (Healthcare Recipient Triad 09)

Alex had taken the time to fully explain the pros, cons and statistics about Covid and vaccination. These were things that had previously worried Lee. (Carer Dyad 03)

In summary, this theme demonstrates participants' orientation that relieving or minimising the initial, unwanted emotions recipients and carers experience in relation to an encounter with a new provider is constitutive of good relationship development, enabling recipients to feel comfortable disclosing personal information and returning to the provider in the future. This theme further demonstrates that the same provider approaches can work to relieve unwanted emotions for, and thereby develop relationships with, recipients and carers.

## 4 DISCUSSION

Using a story completion approach, this study aimed to understand how healthcare recipients and informal carers conceptualise good professional caregiving relationships. Participants' stories were shaped by an overarching discourse that seeking help from, and developing relationships with, new providers can elicit a range of unwanted emotions for both recipients and carers. Relieving or minimising these emotions was seen as central to developing good relationships. A key, and potentially novel, finding demonstrated by the analysis was that participants oriented to these initial, unwanted emotions as stemming not only from fears about recipients' health but from fears that healthcare providers would invalidate recipients' presenting problems and concerns (e.g., that recipients and carers would be ‘made to feel like a nutcase’). This anticipated provider judgement and invalidation was present across a range of stories, therefore likely reflecting how many people, regardless of clinical condition or care setting, feel when embarking on a relationship with a new provider. We expand on this key finding below and explicate additional ways in which this study builds on existing research on professional caregiving relationships.

### 4.1 Conceptualising and enacting relationships beyond disciplines, settings and conditions

Previous research has demonstrated that healthcare recipients can experience anxiety or fear when engaging in help‐seeking with new or existing providers; however, the cause of these emotions is either not explicated^53^, ^54^, ^55^ or is attributed only to the person's presenting problem (e.g., fears about surgical procedures).^56^, ^57^ Previous studies have rarely discussed whether these fears or anxiety might stem from anticipated interactions with providers, as our findings have demonstrated. An exception is work exploring professional caregiving relationships in the context of stigmatised conditions. For instance, Dang et al.^58^ identified that people living with human immunodeficiency virus (HIV) experienced vulnerability and anxiety when beginning a relationship with a new provider. Dang et al.^58^ argued that this anxiety stemmed primarily from the stigmatising nature of HIV and recipients' concerns they would be judged by providers. Our findings similarly show participants' orientation to possible provider judgement or invalidation, however, this was evident in stories focusing on a range of clinical conditions, as well as healthcare disciplines and care settings. Our findings, therefore, demonstrate that anticipated provider judgement and invalidation is not necessarily unique to stigmatised conditions but likely reflects how many people, regardless of clinical condition, feel when embarking on a relationship with a new provider.

The approaches outlined here for relationship development (e.g., small talk, getting to know the recipient, allowing the recipient space to talk, recipient choice) have been identified in existing literature relying primarily on theoretical, researcher and provider perspectives.^30^, ^33^, ^59^, ^60^ Unlike the findings presented here, the existing literature has not specifically or consistently identified these strategies as useful for ensuring recipients and carers are comfortable with the provider and the encounter, highlighting a potentially important point of departure between recipient/carer and provider perspectives. Where previous research has noted the importance of relieving emotions such as anxiety or fear, the ways this can be achieved have either not been explored or have been vague (e.g., through trust, rapport and engagement).^53^, ^54^, ^55^, ^56^, ^61^, ^62^ Notable exceptions are studies exploring chronic, long‐term or stigmatised conditions,^58^, ^63^ however, the strategies identified are often specific to the presenting condition and there is minimal, if any, focus on relieving unwanted or undesired emotions that carers might be experiencing. Our work builds on this literature by demonstrating how relationship development, with both recipients and carers, might be achieved regardless of the nature of recipients' clinical conditions.

Our findings therefore suggest that there might be a set of core behaviours (outlined in Table 2) that healthcare providers across disciplines, care settings and clinical conditions could employ to develop good relationships with recipients and carers. This has implications for interprofessional education and multidisciplinary care delivery, enabling different healthcare disciplines, specialties and teams, to work from the same understanding of relationship development.

### 4.2 Conceptualising and enacting relationships beyond the dyadic and static

Our findings demonstrate participants' orientation to the temporality of professional caregiving relationships. That is, participants' orientations to (1) anticipation (e.g., expecting to be treated poorly by providers), (2) previous healthcare encounters and their impact on subsequent (i.e., delayed) help‐seeking and (3) future encounters (e.g., recipients feeling comfortable to return to providers), demonstrated that they conceptualised relationships in time‐bound ways. The impact of previous negative healthcare encounters on future help‐seeking has been identified in the literature.^64^ We add to this work by understanding this finding in relation to the conceptualisation and possible enactment of professional caregiving relationships. To participants, relationships did not occur in a vacuum but were reflective of a broader passage of time, impacted by a range of prior encounters and relationships as well as influencing future ones. This notion of temporality demonstrates the importance of moving beyond dyadic, static understandings of professional caregiving relationships to recognising the broader care networks and experiences, as well as biographical and temporal contexts, in which these relationships exist.^14^ Importantly, expanding our understanding of relationships in this way does not necessarily require providers to do more within their interactions with recipients and carers. Instead, moving beyond dyadic, static conceptualisations helps providers to understand the relevance of engaging in certain behaviours (e.g., enabling choice), and the potential positive impacts of these behaviours on the immediate healthcare encounter as well as recipients' subsequent help‐seeking practices.

Recognising the impact of broader care networks and relationship experiences reinforces calls for the adoption of relationship‐centred approaches to care that emphasise interdependency and reciprocity rather than dyadic exchanges.^4^, ^38^, ^65^, ^66^ Participants' stories contextualised the characters' help‐seeking experiences and practices within their broader relational and social worlds, where different professional caregiving relationships inevitably influenced each other. However, recipients were also positioned in more active roles than were carers, with carers typically on ‘stand‐by’ and enacting their role primarily in relation to provider‐recipient interactions (e.g., being ready to intervene if the recipient was experiencing communication difficulties). This relative positioning could be seen to align with (1) individualistic understandings of care delivery where the focus is primarily on recipients, and (2) dyadic approaches that focus on provider–recipient interactions only. However, the fact that participants' stories focused on the importance of carers, as well as recipients, feeling comfortable and at ease, meant that carers were arguably seen as integral to professional caregiving relationships. Furthermore, carer characters' initial, unwanted emotions often centred on their concerns for recipients; it, therefore, follows that observing the recipient as comfortable and at ease, rather than having more active involvement, enabled carers to develop good relationships. Hence, the relative positioning of recipients and carers within participants' stories likely reflects recognition that relationship development inevitably requires recipients and carers to occupy different spaces, with the role of carers requiring flexibility depending on how providers and recipients are interacting. Our findings therefore contribute to an understanding of the role of informal carers in professional caregiving relationships, including how their role is positioned in relation to recipients and how they might best be engaged as partners in care delivery.

### 4.3 Strengths and limitations

The strengths of our study are the inclusion of healthcare recipient and carer perspectives and the richness and depth of the data collected, moving the study of professional caregiving relationships beyond methodological individualism.^39^ However, there are some limitations. First, a focus on conceptualisations drawn from hypothetical stories might limit how we can extrapolate from the findings to real‐life healthcare encounters, limiting the study's practical relevance.^44^ However, the discourses people draw on to make sense of the world do not exist in isolation; they reflect broad patterns of understanding that influence how people should, can and do act.^43^, ^67^ Hence, the conclusions drawn from this work are useful for strengthening our understanding of how the stakeholders involved in professional caregiving relationships perceive these relationships, including what is required to develop them. Second, we focused on relationships involving only one healthcare provider. However, participants' stories routinely oriented to the relevance of broader care networks and their relationships with previous providers, indicating that the findings might hold relevance for relationships involving multiple providers. Finally, participants' stories focused predominantly on adult recipients who were able and willing to engage in interaction with minimal support. Relationships (and particularly the role of carers in these relationships) might look different when recipients require significant support to engage (e.g., when they have communication difficulties or cognitive impairment, are young children, or are critically unwell).

### 4.4 Future directions

Future studies might usefully explore conceptualisations of relationships that involve (1) multiple healthcare providers from different disciplines and (2) recipients with varying willingness and ability to engage, further moving conceptualisations of professional caregiving relationships away from simple, dyadic understandings. Efforts must then be made to translate these conceptualisations into evidence‐based teaching and professional development resources. Together, this research will generate much‐needed evidence to inform our understanding of the roles of key stakeholders in relationship development and maintenance and how these roles should be enacted in practice to facilitate positive recipient, carer and provider outcomes.

## 5 CONCLUSION

This study demonstrates how healthcare recipients and informal carers conceptualise high‐quality professional caregiving relationships. Help‐seeking from new healthcare providers was routinely oriented to by participants as eliciting a range of unwanted emotions for recipients and carers, either due to the presenting problem or to the anticipated (potentially negative) interaction with the provider. It was through relieving or minimising these emotions, and recipients' and carers' subsequent feelings of ease and comfort, that good professional caregiving relationships were seen to develop. We have contributed to the literature by demonstrating how professional caregiving relationships might be developed across contexts and disciplines. The findings suggest that the development of good, high‐quality relationships might be achieved via a set of core behaviours that transcend disciplinary, context and condition‐specific boundaries.

## AUTHOR CONTRIBUTIONS

**Rebecca Feo**: Study conceptualisation; data collection; data analysis; writing—original draft preparation; writing—review and editing. **Jessica A. Young**: Data analysis; writing—original draft preparation; writing—review and editing. **Kristy Urry**: Study conceptualisation; data collection; data analysis; writing—review and editing. **Michael Lawless**: Data analysis; writing—review and editing. **Sarah C. Hunter**: Data analysis; writing—review and editing. **Alison Kitson**: Study conceptualisation; writing—review and editing. **Tiffany Conroy**: Study conceptualisation; data analysis; writing—review and editing.

## CONFLICT OF INTEREST STATEMENT

The authors declare no conflict of interest.

## ETHICS STATEMENT

This study received ethical approval from the Flinders University Human Research Ethics Committee Low Risk Panel (ID: 4610). Participants completed an electronic consent form before participating.

## Data Availability

The data that support the findings of this study are available on request from the corresponding author. The data are not publicly available due to privacy or ethical restrictions.
